# Supplementary material for: Geospatial variation in dietary patterns and their association with heart disease in Bangladeshi population: Evidence from a nationwide survey
Source: PLoS One. 2024 Jul 18;19(7):e0307507. doi: 10.1371/journal.pone.0307507 (PMC11257343; doi:10.1371/journal.pone.0307507)
Supplement: S1 Table — (PDF) [file pone.0307507.s001.pdf]

S1 Table. Food items and food groupings.

Food groups	Food items
**Rice**	Rice coarse, Rice medium, Rice fine
**Processed rice**	Beaten rice, Poop rice, Puffed rice
**Biscuits/prepared foods**	Bread, Bonruti, Vermicelli, Suji, Biscuits, Cake
**Other grains**	Other (Food grains)
**Pulse/legumes**	Green gram (boot), Lentil (musur), Chickling-Vetch (mug), Pea gram (kheshari), Mashkalai, Other (pulses)
**Wheat or flour**	Wheat (atta), Flour
**Fish**	Hilsha, Rhui/ Katla/ Mrigel/ Kali baush, Pangash, Boal/ Air, Magur/ Shing, Koi, Silver carp/ Grass carp/ Miror carp, Shoal/ Gajar/ Taki, Puti/ Big Puti/ Telapia/ Nilotica, Mala-kachi/ Chala-chapila/Khalsha, Other small fishes (with Tangra), Shrimp, Dried fish, Eel fish, Sea fish, Baila, Other (fish)
**Meat**	Beef, Mutton, Sheep, Buffalo, Hen, duck, Other (meat)
**Eggs**	Hen eggs, Duck eggs, Other (egg)
**Fruits**	Ripe banana, Mango, Melon/ Bangi, Jack fruit, Leeches, Ripe papaya, Guava, Pineapple, Safeda, Palm, Bedana, Apple, Grape, Orange, Black berry, Amra/Kamranga, Other (fruits)
**Non-starchy vegetables**	White gourd/ Pumpkin, Brinjal, Water gourd, Balsam apple, Perbol (Patal), Snake gourd/ Ribbed gourd, Green Banana, Cauliflower/ Cabbage, Bean/ Lobey, Tomato, Radish, Ladies' finger
**Starchy vegetables**	Potato, Arum/ Ol-kachu/ Kachur-mukhi
**Leafy vegetables**	All types of leafy vegetables (Spinach/ Amaranta/ Basil)
**Other vegetables**	Other (vegetables)
**Dairy**	Liquid milk, Powder milk, Curd, Casein (ponir)/ Butter, Milk drinks, Other (Milk and Dairy)
**Pickles**	Pickles, Amsatta

**Fast foods**	Fried chicken, Patties/Cake, Sandwich, Burger, Hotdog, Pizza
**Jam and jelly**	Jam, jelly
**Sauce and sirka**	Sauce, Sirka
**Outside meal**	Briyani,/rice, Fish, Meat
**Deep-fried snacks**	Samucha, Singara, Puri
**Fats and oil**	Soybean oil, Mustard oil, Palm oil, Ghee, Dalda/ Vanashpati, Other (oil and fats)
**Salt**	Salt
**Spices**	Dried chili, Green chili, Onion, Garlic, Turmeric, Garlic, Cummin, Coriander-seed, Aromatic-seed, Clove/ Black pepper/ Cassia-leaf, Other (Spices)
**Carbonated drinks**	Carbonated soft drinks (Pepsi/RC/Mojo/Coke, Sherba), Soft drinks
**Non-carbonated drinks**	Tea, Coffee, Tea/ Coffee leaf, Ovaltine/ Horlicks, Liquid (Ros) of Sugarcane/ Date/Palm, Green coconut water
**Sugar and sweetmeat**	Rasogolla, Chamcham, Shandash, Jilapi, Bundia, Amriti, Halua, Batasha, Kadma, Khaja/ Logenze/ Toffee, Chocolate, Ice-cream, Sugar, Misri, Molasses (Sugarcane/ Date/ Palm), Other (sweetmeat)
