# Supplementary material for: Geospatial variation in dietary patterns and their association with heart disease in Bangladeshi population: Evidence from a nationwide survey
Source: PLoS One. 2024 Jul 18;19(7):e0307507. doi: 10.1371/journal.pone.0307507 (PMC11257343; doi:10.1371/journal.pone.0307507)
Supplement: Attachment — Submitted filename: Response to Reviewers.docx [file pone.0307507.s002.docx]

July 4, 2024

**Re: Response to reviewers on manuscript [PONE-D-23-21261R1]**

Dear Editor,

I hope this message finds you well. I appreciate the constructive feedback provided by the respected reviewers on our manuscript titled “Geospatial variation in dietary patterns and their association with heart disease in Bangladeshi population: Evidence from a nation-wide survey.” We have carefully considered each comment and have made revisions accordingly. Please find below our detailed responses to each point raised:

**Reviewer #4: Thanks for the beautiful work for Bangladesh people, Please work on the following points:**

1. **Comment:** How do you proof you received the data, what is the document states that you got the data?

**Response:** Thank you for your concern. We purchased the datasets from the Bangladesh Bureau of Statistics (BBS). Md Ruhul Amin and Md Masum Ali, two of our co-authors, were engaged in this process. To verify our data acquisition, we have attached the payment receipt.

2. **Comment:** It is not so good to apply a survey result which is done in 2016 for your people 2024, how curiously they read the article and accept the results?!. Where not any new data? If not, why did not you collected a newer version? You could choose some criteria that is suitable and allowed from the your local government.

**Response:** Thank you for your insightful comments. We understand the concern regarding the use of a 2016 dataset for a study conducted in 2024. Unfortunately, there are no more recent datasets available that meet the necessary criteria for this research. Despite the dataset being from 2016, it remains highly relevant. It provides valuable insights into the topic under investigation, and the change of dietary patterns at the population level takes a longer period. The authors thought that it was okay to answer research questions posed in this paper using this data set. Further, we ensured rigorous analysis and interpretation to draw meaningful conclusions that can still be applicable today. Additionally, the 2016 dataset is comprehensive, and national representative, making it a suitable foundation for our study. We hope this addresses your concerns and highlights the significance of our findings.

3. **Comment:** Line 127 Variables of the study, the section of education is too wide since higher levels of education result in better knowledge of keeping self against health problems.

**Response:** Yes, I agree with you. Therefore, education was adjusted to see the effects of dietary patterns on heart disease. Further, the literature supports the inclusion and categorization of education variables for this study. The category used in this paper is widely used in Bangladesh, as can be seen in the following references of our manuscript (29, 32):

1. Sarwar N, Ahmed T, Hossain A, Haque MM, Saha I, Sharmin KN. Association of dietary patterns with type 2 diabetes mellitus among Bangladeshi adults. International Journal of Nutrition Sciences. 2020 Dec 1;5(4):174-83. doi: [10.30476/IJNS.2020.88173.1091](https://doi.org/10.30476/ijns.2020.88173.1091)
2. Khanam F, Hossain MB, Mistry SK, Afsana K, Rahman M. Prevalence and Risk Factors of Cardiovascular Diseases among Bangladeshi Adults: Findings from a Cross-sectional Study. J Epidemiol Glob Health. 2019 Sep;9(3):176-84. doi: 10.2991/jegh.k.190531.001
4. **Comment:** This is not nice to put link in the middle of an article like in line 191 online(<https://data.humdata.org/dataset/cod-ab-bgd>) using Arc GIS 10.8.1 where geospatial... Please make a reference to it.

**Response:** Thank you for your feedback. We removed the link from the middle of the article and added it to the references section instead, which can be found in line 217. The added reference is given below:

OCHA. Bangladesh - Subnational Administrative Boundaries; 2020 [cited 2024 Jun 12]. Available from: <https://data.humdata.org/dataset/cod-ab-bgd>

5. **Comment:** Lines 216-224 does not need bold and link, smoothly talk about figure 2.

**Response:** Thank you for your nice feedback. In the revised manuscript, we removed the link, which can be found on lines 244-248. Furthermore, the bolded line is the title of the figure. According to the PLOS ONE submission guidelines, “**each figure caption should appear directly after the paragraph in which it is first cited, with the figure title in bold. The figure legend should follow the title in normal text”.** Therefore, to adhere to these guidelines, we bolded the figure titles.

6. **Comment:** While the data collected in Bangladesh, it does not need to mention all the time in Bangladesh like in the table heads and section heads.

**Response:** Thank you for your nice comment. We agree with you. Now, in the revised manuscript, we removed “in Bangladesh” from tables and section heads to prevent the overuse of the phrase.

7. **Comment:** Please move line 267 Table 4. Characteristics of the study participants by tertile categories of dietary patterns. to the table page.

**Response:** Thank you. This has been moved to the second revised manuscript as can be seen in line 288.

8. **Comment:** Once again, lines 277 and 286-292 is bold and only need to mention the figures easily.

**Response:** Thank you for your suggestion. The bolded lines (298, 306) are the title of the figures. According to the PLOS ONE submission guidelines, “**each figure caption should appear directly after the paragraph in which it is first cited, with the figure title in bold. The figure legend should follow the title in normal text.”** Therefore, to adhere to these guidelines, we bolded the figure titles.

9. **Comment:** The conclusion section is too short.

**Response:** Thank you for your insightful comment. The conclusion has been rewritten in the revised version.

10. **Comment:** I would like you to suggest some solutions and methods to your people in Bangladesh to protect themselves from heart diseases and add suggestions in the discussion or conclusion part.

**Response:** Thank you for your suggestions. Here, we think that you might examine the first submission of the manuscript. However, during the first revision, we addressed this issue. We added some suggestions/solutions and policy implications for Bangladeshi people, which can be found on lines 423-431:

“This study identified the prevailing dietary patterns across the country, providing valuable insights for the government to develop effective guidelines for tailoring heart disease prevention programs. Our study recommends a public awareness campaign to promote a heart-healthy dietary choice, such as incorporating rice, vegetables, fruits, and spices, while discouraging the consumption of fast food and sugary items through mass media, educational institutions, and other channels. Furthermore, the government should impose a tax on unhealthy food items and provide subsidies on heart-healthy food items to ensure affordability for the general population. As this study identified the vulnerable regions to heart disease and unhealthy dietary patterns, it can provide valuable insights for policymakers for tailoring region-specific interventions.”

11. **Comment:** Please add the DOI link or website link for reference number 16, 25, 26, 32, 37, 38, 39, 43, and 46 to maintain the uniformity of the references.

**Response:** Thank you for your suggestion. In the revised version, we have followed the guidelines of PLOS ONE for references. We included the URLs of the references where appropriate and possible, according to the PLOS ONE guidelines. Some of the references you spotted are books or book sections, which have been formatted according to the PLOS ONE guidelines, and these guidelines do not require a DOI or website link for such references. Furthermore, for published articles without DOIs, the PLOS ONE guidelines indicate that they should be presented without a DOI or link.

12. **Comment:** Most of the references look like they have been brought from local authors, please add some major references especially the new ones, after 2022.

**Response:** Thank you for your constructive feedback. In the introduction section, we provided the available evidence on related topics in Bangladesh to identify the research gap. In the discussion, we compared the findings of our study with earlier studies conducted in Bangladesh, which resulted in the inclusion of several studies from local authors. However, following your suggestion, we have carefully reviewed the literature and added some recent related references from the literature. The following references are added (1, 15, 22, 48, 50, 53):

1. Martin SS, Aday AW, Almarzooq ZI, Anderson CA, Arora P, Avery CL, et al. 2024 Heart Disease and Stroke Statistics: A Report of US and Global Data From the American Heart Association. Circulation. 2024 Feb 20;149(8):e347-e913. [doi: 10.1161/CIR.0000000000001209](https://doi.org/10.1161/CIR.0000000000001209).
2. Wei L, Fan J, Dong R, Zhang M, Jiang Y, Zhao Q, et al. The Effect of Dietary Pattern on Metabolic Syndrome in a Suburban Population in Shanghai, China. Nutrients. 2023 May 4;15(9):2185. doi: [10.3390/nu15092185](https://doi.org/10.3390/nu15092185)
3. Proma AY, Das PR, Akter S, Dewan SMR, Islam MS. The urgent need for a policy on epidemiological data on cardiovascular diseases in Bangladesh. Health Sci Rep. 2023 Jul 7;6(7):e1410. doi: 10.1002/hsr2.1410
4. Ahmed W, Muhammad T, Maurya C, Akhtar SN. Prevalence and factors associated with undiagnosed and uncontrolled heart disease: A study based on self-reported chronic heart disease and symptom-based angina pectoris among middle-aged and older Indian adults. PLoS One. 2023 Jun 28;18(6):e0287455. doi: 10.1371/journal.pone.0287455
5. Ram S, Chandra R, Kundu A, Singh A, Singh S, Tanti A, Bhattacharjee B, Tripathi P. Prevalence and determinants of self-reported heart disease among Indian men aged 15–54 years: Evidence from NFHS-5. Clinical Epidemiology and Global Health. 2023 Sep 1;23:101374.
6. Islam S, Nowar A, Amin MR, Shaheen N. Cost of Recommended Diet (CoRD) and Its Affordability in Bangladesh. Foods. 2023 Feb 13;12(4):790. doi: 10.3390/foods12040790

Thank you very much for your co-operation. Please let me know if you have any queries.

Sincerely,

Md. Ruhul Amin

Professor

Institute of Nutrition and Food Science

University of Dhaka, Dhaka, Bangladesh

Phone: +8801819504434

ruhul.infs@du.ac.bd
